# Southern Field and Fireside

**Published:** 1859-08

**Authors:** 


SOUTHERN FIELD AND FIRESIDE.

We have received several numbers of the *Southern Field and Fireside*, and hail its advent with great pleasure as another illustration of the true mode of establishing southern independence.

The *Southern Field and Fireside* is published in Augusta, Ga., and is a most admirable Weekly, devoted to agriculture, literature, and art, and we confidently hope, that this enterprise will attract a large portion of "that vast stream of southern money that flows perpetually northward, to sustain northern literature."

Its agricultural department is under the charge of Dr. Daniel Lee, the distinguished Professor of Agriculture in the University of Georgia. The literary editor is Mr. W. W. Mann, of Augusta, an accomplished writer, and was for several years, the Paris Correspondent, of the National Intelligencer and Southern Literary Messenger. The horticultural department is under the management of Mr. Wm. N. White, a skillful and experienced cultivator of fruits, flowers and vegetables—a writer of repute in these departments, and author of that popular work, "Gardening for the South." It will be as it proposes, a first class paper, and as such we recommend it.

It is in Quarto form of eight pages, folio size, each issue containing forty columns of matter. It is furnished to subscribers at the low price of $2 per annum.

On matters pertaining to their respective departments, address the editors. On matters of business generally, address the publisher, James Gardner, Augusta, Ga.

